# Preeclampsia and Related Cardiovascular Risk: Common Genetic Background

**DOI:** 10.1007/s11906-018-0869-8

**Published:** 2018-07-03

**Authors:** Michalina Lisowska, Tadeusz Pietrucha, Agata Sakowicz

**Affiliations:** Department of Medical Biotechnology, Medical University of Lodz, Zeligowskiego 7/9, Łódź, Poland

**Keywords:** Preeclampsia, Pre-eclampsia, Pregnancy, Cardiovascular disease, Cardiovascular risk, Genetics

## Abstract

**Purpose of Review:**

Preeclampsia (PE) is a hypertensive disorder exclusive for pregnancy. It affects women all over the world and poses a great threat to life, both for mother and child. No definitive treatment exists and placenta delivery comprises the only known cure for PE. One of the most severe complications observed in preeclamptic women is the occurrence of cardiovascular diseases (CVDs) later in life.

**Recent Findings:**

Both PE and CVDs share some of their pathogenic pathways and gene variations. Thus far, a number of publications have examined those relationships; however, almost all of them focus only on common risk factors. The precise pathomechanism and genetic basis of PE and its associated cardiovascular complications remain unknown.

**Summary:**

Therefore, the aim of this review is to unify and clarify the current state of knowledge and provide direction for future studies, especially those regarding the genetic aspect.

## Introduction

Hypertension and its related anomalies constitute one of the most frequent complications of pregnancy. A systematic analysis by the WHO published in 2014 places hypertensive disorders among the direct causes of perinatal death in women [1]. A stepwise reduction in maternal mortality has been observed over the last 25 years, falling almost 44% globally between 1990 and 2015 [2]. Nonetheless, despite the undertaken efforts, there is still a broad spectrum of life-threatening disorders which has not been managed. One such example is preeclampsia (PE), a clinical syndrome known for centuries, but remains incurable [3]. This hypertensive disease is associated with long-term vascular and metabolic alterations, influencing cardiovascular risk later in life. Regardless of their relationship with PE, cardiovascular diseases (CVDs) continue to be leading causes of global mortality [1, 4]. It was estimated for the US population that almost 40% of women and more than half of men free from CVD at the age of 50 years will develop CVD in later life [5]. Globally, it is predicted that two of the most common CVDs, stroke and ischemic heart disease, will be the top two leading causes of death in 2030 [6].

Although the relationship between CVDs and PE has been studied many times, the majority of available articles focus on the risk factors common for both disorders. Thus far, a number of studies implying probable polymorphisms and gene candidates related to PE and CVDs have been published. Nonetheless, only a few of them are genome-wide studies or meta-analyses, assessing the influence of multiple samples and data sources. Therefore, the objective of this review is to gather recent data from the area of pathogenesis and the possible genetic changes underlying PE and its cardiovascular alterations to pave the way for future studies.

## The Association of Preeclampsia and Future Cardiovascular Risk

**Fig. 1:**
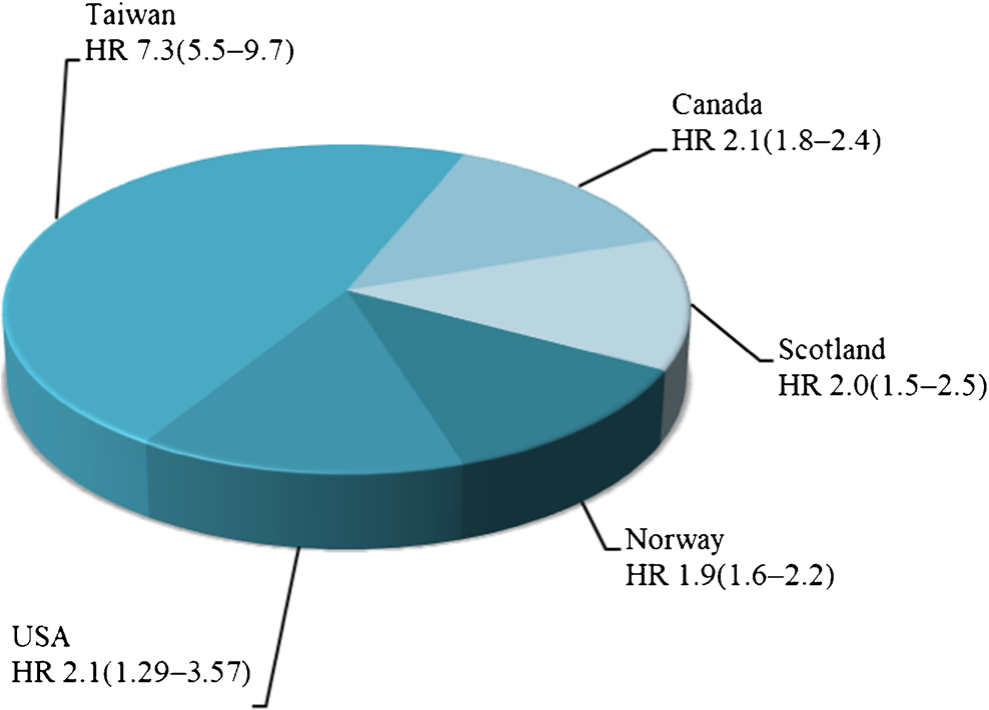
Population-specific risk of cardiovascular disease or death in woman with pregnancy complicated by preeclampsia. HR- hazard ratio

Current literature thoroughly describes the connection between PE and related cardiovascular alterations, and numerous publications examine the common risk factors between them. Most list obesity, hypertension, hyperlipidemia, diabetes mellitus or physical inactivity as the main risk factors [7, 8, 9••, 10, 11]. The general risk of the development of cardiovascular alterations in women with prior hypertensive disease of pregnancy varies according to population. Some of the population-specific risk variations are presented in Fig. 1 [12]. According to Ahmed et al., globally, the most frequent manifestation of vascular disease is stroke; the risk of its development is increased 14.5-fold after PE [95% confidence interval (CI) 1.3–165.1]. In contrast, angina stands as the rarest complication, with a hazard ratio (HR) of 1.53 (95% CI: 1.09–2.15) [13].

Some studies suggest that hypertensive pregnancy itself does not influence cardiovascular alterations, but some pre-existing risk factors may in fact determine later cardiovascular risk [8]. A similar conclusion was reached by Bellamy et al. who suggests the presence of a possible pathological phenotype in women with recurrent PE, putting them at risk of hypertension and CVDs [9]. In contrast, Hromadnikova et al. propose that CVDs in later life may develop as a result of undergoing epigenetic changes related to complicated pregnancy [14•].

Despite the wealth of acquired data, some questions remain unanswered: Whether PE pathogenesis causes cardiovascular alterations, whether PE develops as a result of previously hidden cardiovascular abnormalities or whether the risk of both diseases is linked with factors existing before pregnancy. To clarify these points, it is necessary to learn more of the pathogenesis of the disease and its underlying genetics.

## Preeclampsia Pathophysiology: Overview

PE is a complex disorder exclusive for pregnancy. The diagnosis criteria comprise recognition of the new onset of hypertension with coexisting proteinuria [15]. Because PE has a heterogeneous nature, its exact pathomechanism remains unclear; however, there are many theories addressing its behaviour. Most of them list oxidative stress, immunologic intolerance and angiogenic imbalance as the main causes [3, 16]. Despite the diversity of these theories, all share one common item, the placenta. The placenta is known to play a central role in PE pathogenesis as its presence is required for the development of the disease, and delivery of the placenta represents the only known cure [16].

The most important factors observed in the development of PE are inadequate trophoblast invasion and improper spiral artery development. In consequence, low-resistance vessels are not formed, leading to decreased blood flow and insufficient supply to the fetus. Eventually, these events trigger a restriction in uteroplacental circulation and result in an insufficiently perfused, ischemic and hypoxic placenta. As a consequence, local oxidative stress results in systemic inflammation, and the release of pro-inflammatory cytokines [12, 17]. These changes cause endothelial dysfunction, which manifests itself as increased vascular permeability. Junction widening allows immune cells to infiltrate the vascular wall and exacerbate existing inflammation [18, 19]. Endothelial malfunctions are also observed in the kidneys: this specific glomerular lesion is known as ‘glomerular endotheliosis’ and it is associated with existing proteinuria [20].

Although abnormal utero-placental perfusion plays a significant role in the development of PE, some other maternal factors may significantly modulate the risk of this disease. Two of the most common subtypes of PE are known as early-onset PE and late-onset PE. Early-onset PE appears before the 34^th^ week of pregnancy and is usually manifested by low birth weight and intrauterine growth restriction (IUGR) and is believed to be of placental origin. In contrast, late-onset PE (≥ 34^th^ week) is generally related to maternal conditions such as obesity, diabetes or chronic kidney diseases [21–23]. The same clinical factors play a significant role in the development of CVDs [7, 8, 9••, 10, 11, 24].

Moreover, there is strong evidence that PE is connected with familial predispositions. A higher chance of PE has been found in the daughters of preeclamptic women during their own pregnancies [25]. In addition, a woman whose partner has previously fathered a preeclamptic pregnancy with another woman is also at higher risk of PE, implying that paternal genetic factors also play a role [26]. However, the inheritance effect along the maternal line is visibly stronger than the paternal one: 2.2 vs. 1.5, respectively [27].

## The Association Between Preeclampsia Pathogenesis and Cardiovascular Disease

**Fig. 2:**
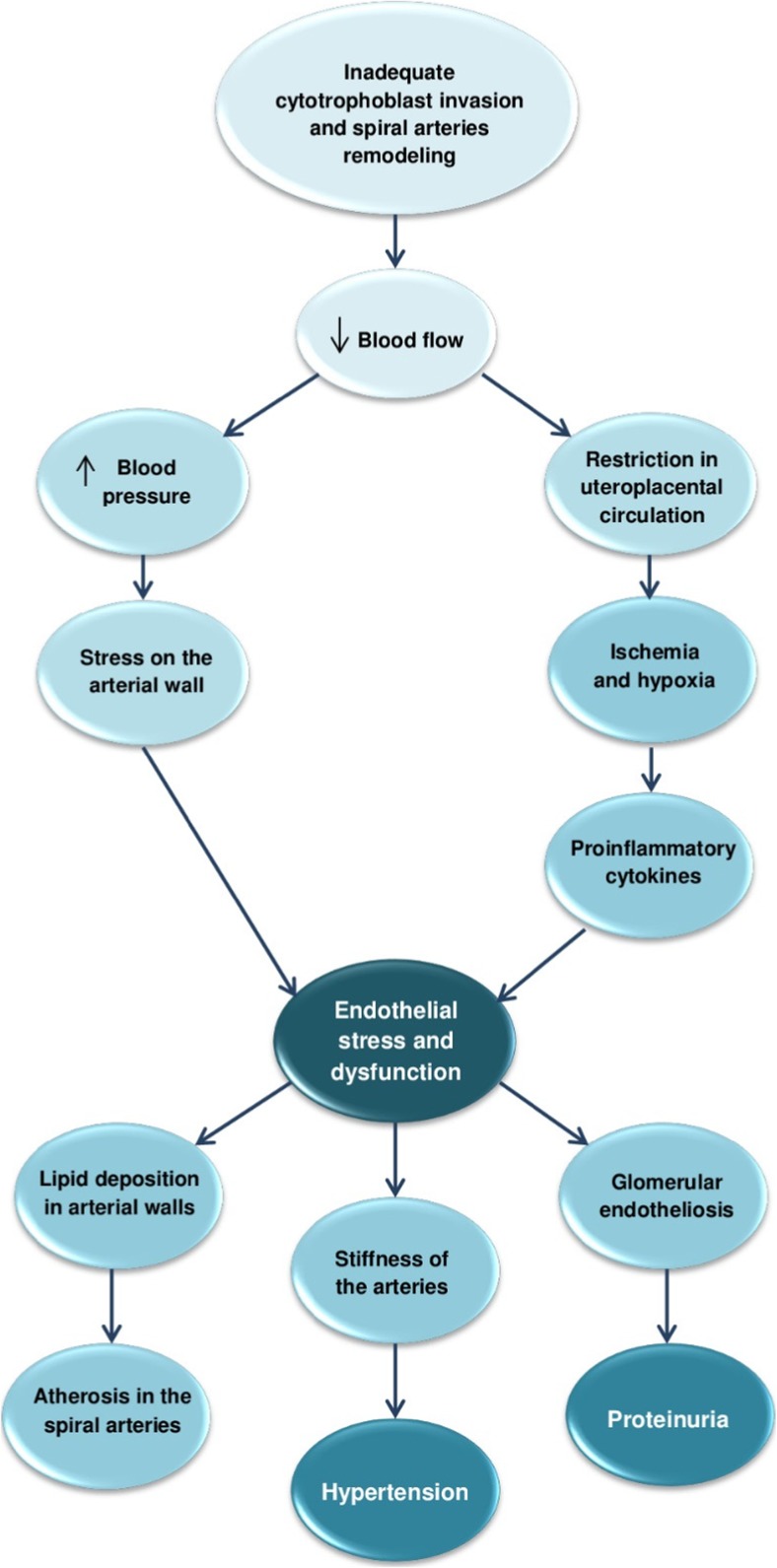
Common pathogenic pathways for preeclampsia and preeclampsia-related cardiovascular alterations. Decrease ↓ ; increase↑

Preexisting vascular endothelial dysfunction is currently viewed as a key common factor shared between PE and PE-related cardiovascular alterations. The elevated blood pressure typical for both medical conditions causes shear stress on the arterial wall, which promotes endothelial dysfunction and stiffness of the arteries. Moreover, dysfunction of the endothelium in the vessels is considered as an initial manifestation of arterial ageing [28]. A brief summary of such pathological changes is outlined in Fig. 2.

Inadequate spiral artery remodeling is one of the key characteristics of PE. During the development of PE, insufficient decidual arterial modification can result in their vasculopathy or acute atherosis. The former is characterized inter alia by foam cell infiltration into the vessel walls and subsequent constriction and high resistant flow. This results in elevated maternal diastolic blood pressure. Additionally, preeclamptic women diagnosed with decidual vasculopathy exhibit higher risk of chronic hypertension [29]. The latter, acute atherosis, develops solely in the spiral arteries. A possible explanation for its pathogenesis can be found in changes taking place in the uteroplacental circulation. Constricted, highly resistant vessels generate high flow velocity which may damage the villous surface architecture. Additionally, smooth muscle cells retained in improperly formed arteries escalate the hazard of spontaneous vasoconstriction and recurring perfusion of the intervillous space, generating ischemia- reperfusion injury [30, 31]. As a consequence, two other characteristic components of both PE and CVDs are presented: inflammation and endothelial stress. The latter has been suggested to promote lipid deposition in the arterial walls, which may serve as an explanation for the development of acute atherosis in the decidual parts of the spiral arteries [30].

The generation of inflammation and endothelial stress results in enhanced production of pro-inflammatory factors. It was previously described that chronic inflammation may trigger foam cell formation [32]. Elevated levels of pro-inflammatory cytokines are responsible for monocyte and macrophage activation. Activated macrophages internalize lipoproteins, creating foam cells which together with formed fibrotic plaques are the key characteristics of atherosclerosis, one of the most serious cardiovascular complications [33]. Depending on the activation manner, macrophages can be divided into classically (M1) and alternatively activated (M2) groups; the M1 phenotype is regarded as pro-inflammatory, and M2 as anti-inflammatory. The imbalance in the M1-to-M2 ratio may lead to plaque development and impaired inflammatory response [33, 34]. Macrophages also demonstrate distributional changes in PE. However, there is a significant difference in assumptions considering both conditions. It is suggested that atherosclerotic plaque formation includes mainly M2 macrophages, while PE pathogenesis is linked with decreased M2 level [34, 35]. The results of in vitro studies suggest that the presence of activated macrophages may contribute to inadequate trophoblast invasion and improper vessel remodeling [36]. To better understand the role of changes in macrophage phenotype in this area, future investigations are needed.

During PE, placental ischemia induces the production and release of placental agents including angiogenic factors known to be implicated in the progression of endothelial and vascular dysfunction, and subsequent hypertension [37]. Anti-angiogenic FMS-like tyrosine kinase 1 (sFlt-1) antagonizes two important angiogenic agents: vascular endothelial growth factor (VEGF) and placental growth factor (PlGF). sFlt-1 binds to them, thus preventing their interaction with receptors. In vivo studies conducted on pregnant rats demonstrated that sFlt-1 administration induces hypertension, proteinuria and glomerular endotheliosis in animals [38]. Furthermore, the sFlt-1 level was proven to be elevated in preeclamptic patients and to correlate positively with the disease severity [37, 39].

Another widely studied anti-angiogenic factor is soluble endoglin (sEng). sEng and sFlt-1 are believed to be responsible for endothelial dysfunction in vitro. Moreover, like sFlt-1, sEng levels are known to increase with the disease severity [40]. These factors are also related to the risk of CVD. sFlt-1 is linked to cardiovascular remodeling and was demonstrated to be elevated in patients with heart failure. Additionally, it was suggested as a potential biomarker of cardiovascular risk [41]. Similar facts have been established for sEng, as it is considered as a reliable candidate for an indicator of hypertension, hypercholesterolemia, endothelial dysfunction and cardiovascular risk [42, 43].

## Underlying Genetics

Most current studies on the relationship between PE and CVDs are focused on clinical similarities and common risk factors. Based on the changes that take place, and the possibility that increased risk for both disorders may be dependent on pre-pregnancy factors, current studies should address genetic aspects.

The vast majority of the tested genes and polymorphisms are directly related to the alterations observed in the pathological processes underlying both CVDs and PE. One of the largest of such studies was conducted to identify the common transcriptomic signatures involved in PE and CVDs [44]. A novel, bioinformatic meta-analysis investigating gene expression profiles from cardiovascular patient blood samples and placental tissue samples from preeclamptic women by Stiras et al. found that of 181 and 925 genes differentially expressed in CVD and PE, respectively; 22 genes were common for both disorders. Among them were those involved in oxidative stress, inflammation-mediated cytokines and chemokines, interleukin signaling or B-cell activation, all of which are found to take part in the complex pathogenesis of both diseases [12, 17, 45–48].

Some studies emphasize that both PE and CVDs share several lipid metabolism abnormalities. Johansson et al. performed a whole genome transcriptional profiling in decidual basalis tissue from Norwegian preeclamptic cases and controls and in lymphocytes from Mexican-American cases. They found that over 100 genes demonstrate various expression profiles among preeclamptic and control cases. Between those genes, only ACOX2 showed an inverse correlation with triglyceride levels and PE status [49]. Furthermore, they found that one single nucleotide polymorphism (SNP) of the ACOX2 gene (rs4681689) was associated (*p* < 0.05) with triglyceride levels.

ACOX2 is a peroxisomal enzyme physiologically responsible for one of the stages in the degradation of branched-chain fatty acids and bile acid. Down-regulation of its gene, and subsequent ACOX2 deficiency, results in fatty acid and bile acid accumulation [50]. Johansson et al. also found that ACOX2 level was inversely correlated with triglyceride levels, suggesting that down-regulated ACOX2 is associated with increased circulating triglyceride levels [49]. It was previously described that an abnormal lipid profile is associated with increased oxidative stress and endothelial dysfunction [51]. Additionally, high triglyceride levels are well-known CVD risk factors, and were proven to be elevated in preeclamptic patients, together with total serum free fatty acid level [52–55]. Therefore, it was suggested that ACOX2 may be a possible genetic risk factor linking PE and related CVDs [49].

Although changes in the gene expression level are known to be related to PE pathogenesis, some studies have also implicated gene structural changes. Groten et al. assessed five different polymorphisms (Tyr113His EPHX1, 894G/T and 4a4bVNTR eNOS, Met235Thr AGT and –401T/C ESR1) previously found to be associated with CVDs and impaired endothelial function [49]. Blood samples were taken from populations of Caucasian subjects (N = 250) and black African women (N = 220). Of all the tested polymorphisms, only epoxide hydrolase 1 (EPHX1) and variable nucleotide tandem repeats in intron 4 of the endothelial nitric oxide synthase (eNOSI4) were found to have a statistically significant relationship with the course of PE and its development [56].

Microsomal EPHX1 is an enzyme involved inter alia in the regulation of the oxidation status of xenobiotic-derived intermediates [57]. It has also been found to play a protective role against oxidative stress in an in vitro study assessing the influence of EPHX1 on embryo development in coculture [58]. Therefore, it is probable that altered variants of the enzyme disrupt its proper function, thus contributing to the development of PE and CVDs by oxidative stress-mediated and epoxide-related cytotoxic damage. Another significant polymorphism concerns eNOS, an enzyme involved in nitric oxide (NO) synthesis in the vascular endothelium. NO, an important relaxing factor, is implicated inter alia in regulating vascular tone, myocardial contractility and inhibiting platelet aggregation [59]. It was proven in vivo that the altered expression of eNOS, possibly caused by gene polymorphism, results in NO synthesis defects. Moreover, eNOS-deficient mice were shown to develop hypertension, hyperlipidemia and metabolic insulin resistance [60]. As those alterations, together with constricted vessels, are one of the main features of both PE and CVD, it seems plausible that eNOS genetic variants may be relevant to the disease outcome.

Groten et al., report that carriers of the EPHX1polymorphism on exon 3 (Tyr113) demonstrated 3.6- fold increased risk of PE development (95 % CI: 1.366-8.750), and those carrying the eNOSI4 VNTR4a polymorphism demonstrated a 1.7- fold increased risk (95 % CI: 1.105-2.705) [56]. This supports previous research from Finland, in which the EPHX1 polymorphism on exon 3 was reported to be linked with PE (N=248)[61]; however, the odds ratio for PE associated with the Tyr113 polymorphism was 1.61 (95% CI: 1.12 – 2.32); this difference may result from diverse patient populations. Studies conducted on black South African patients and Turkish women further indicate the significant influence of population diversity, as no significant alterations between groups were found in either study [62, 63].In addition, previous studies on the eNOSI4 polymorphism conducted in India and Iran fail to confirm any increased susceptibility of 4a allele carriers to PE [64, 65]. Nonetheless, the presence of differences in obtained outcomes may again suggest an interdependence between the polymorphism and type of population.

Another search conducted in the area was based on previous findings confirming the PE susceptibility locus on chromosome 2q22[66, 67]. This family-based study (Australian family cohort) included testing four different SNPs risk variants on the following genes: lactase (LCT; rs2322659), low density lipoprotein receptor-related protein 1B (LRP1B; rs35821928), rho family GTPase 3 (RND3; rs115015150) and grancalcin (GCA; rs17783344). All of the tested SNPs were found to be associated with PE and known cardiovascular risk factors, such as weight, height, waist-to-hip ratio, blood glucose and triglycerides [68••]. These findings again indicate that CVDs and PE genetic mechanisms are shared, at least to a certain extent.

Systemic inflammation, with the coexisting release of pro-inflammatory molecules, was previously mentioned as a common characteristic for both PE and related cardiovascular alterations. The key factor regulating inflammatory response, and which is implicated in inflammation-associated diseases, is nuclear factor kappa beta (NFkB) [69]. Cytokine-dependent NFkB activation, followed by macrophage mobilization, results in further release of chemokines and cytokines. One of the main factors activating NFkB, and thus the mentioned process, is inhibitory kappa B kinase beta (IKKB/IKK2). However, IKKB and NFkB, known to be implicated in observed systemic inflammation, may also act independently from each other. Chen et al. suggest a dual role of the molecules, both in prevention of the inflammation and in simultaneous increase in local injury [70]. However, the mode of IKKB function is still conflicting, as it may act through diverse targets.

It has already been established that NFkB plays a broad role in CVDs. It was proven in vivo that persistent NFkB activation in mice with heart failure promotes adverse remodeling, endoplasmic reticulum stress and apoptosis [71, 72]. Furthermore, IKK/NF-κB activation in cardiomyocytes was shown to induce excessive inflammatory response and myocyte atrophy, resulting in cardiomyopathy and heart failure [73]. NFkB also plays a prominent role in the pathogenesis of PE. Its placental expression was measured to be nearly 10-fold higher in preeclamptic pregnant women than in healthy pregnancies [74]. Among other things, NFkB possibly influences the pathogenesis of PE by inducing increased trophoblastic apoptosis [75].

As mentioned before, the key NFkB regulator IKKB may act independently of its target molecule. Thus, there is a possibility that its role in PE and CVD pathomechanisms might also be distinct. With this in mind, testing IKKB expression levels in affected patients seems to be an approach worth considering.

## Epigenetics

Not only genetics, but also epigenetics, may yield new insights into the pathogenesis of PE and CVDs. Hromadnikova et al. conducted a retrospective cohort study based on Caucasian pregnant women (N = 160) [14•]. The researchers focused on exploring the microRNA maternal profile in whole peripheral blood, regarding its previously established relationship with cardiovascular and cerebrovascular diseases. The tested groups included pregnancies complicated with gestational hypertension, intrauterine growth restriction (IUGR), PE and normal pregnancies as controls. The findings reveal miR-100-5p and miR-125b-5p to be deregulated in clinically established PE. Additionally, those microRNAs were also found to be deregulated in two other studied groups [14•]. Previous studies confirm a possible association of miR-100-5p with PE. In vivo studies on mice have shown that specific inhibition of miR-100-5p significantly increased expression of IL-6, a pro-inflammatory cytokine known to be elevated in PE women [76, 77]. IL-6 has also been found to play a key role in the pathophysiology of CVDs [45].

Another approach described by Oudejans et al. is based around the epigenetic changes associated with elevated risk of the disease. The study outcome gives a set of 12 differentially methylated regions (DMRs) which may be possibly related to the pathogenesis of CVDs and PE. Moreover, the authors suggest that DMRs identified in this way may serve as a foundation of the future cohort studies [78••].

## Future Directions and Conclusion

PE and PE-related CVD are known to have common pathogenic pathways. Among the numerous pathological changes taking place in both disorders, the most important ones are hypertension, endothelial dysfunction, local inflammation and oxidative stress. The most probable hypothesis of the relationship between the diseases includes the risk factors present before pregnancy, as well as the contribution of later epigenetic changes. Most importantly, the presented review underlines the need for more significant studies based on larger study populations and supplemented by follow ups to allow a more thorough understanding of the pathogenesis of complex PE and PE-related CVDs. Furthermore, it is essential to create specific gene sets dedicated to clearly defined ethnic populations. A holistic, yet population-specific, approach is needed to eventually yield measurable data, translating into disease prevention.
